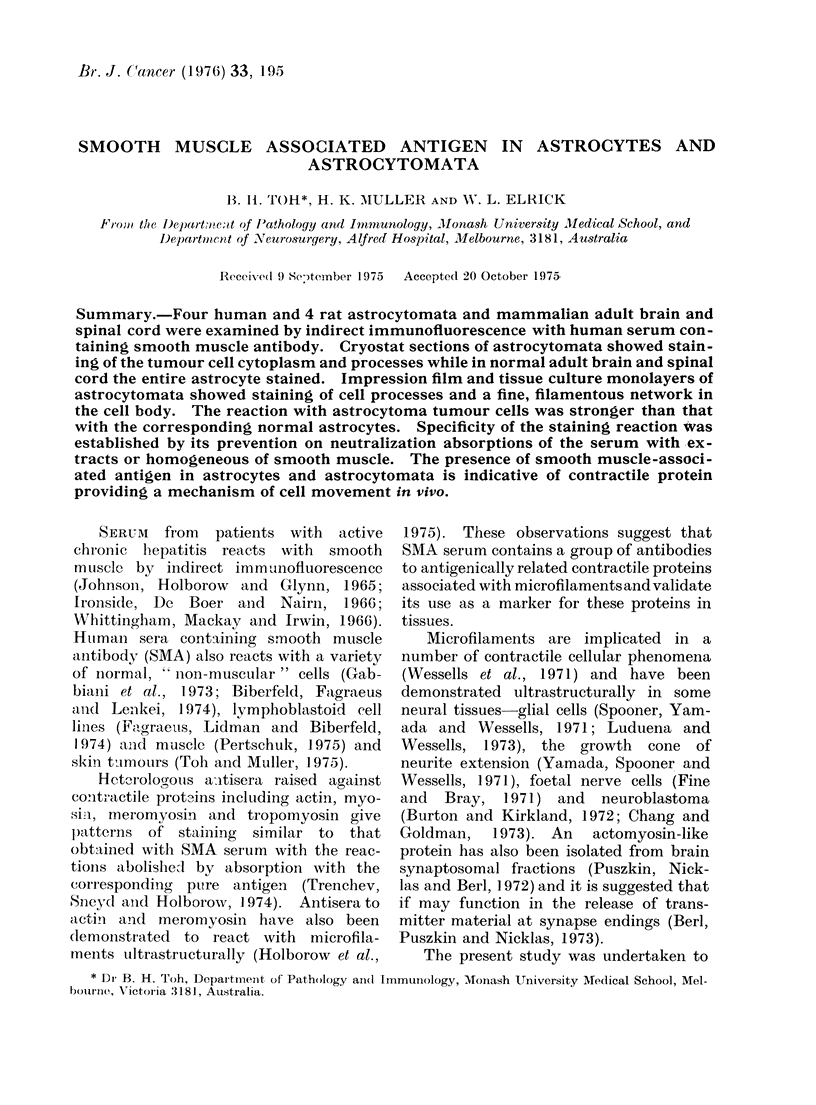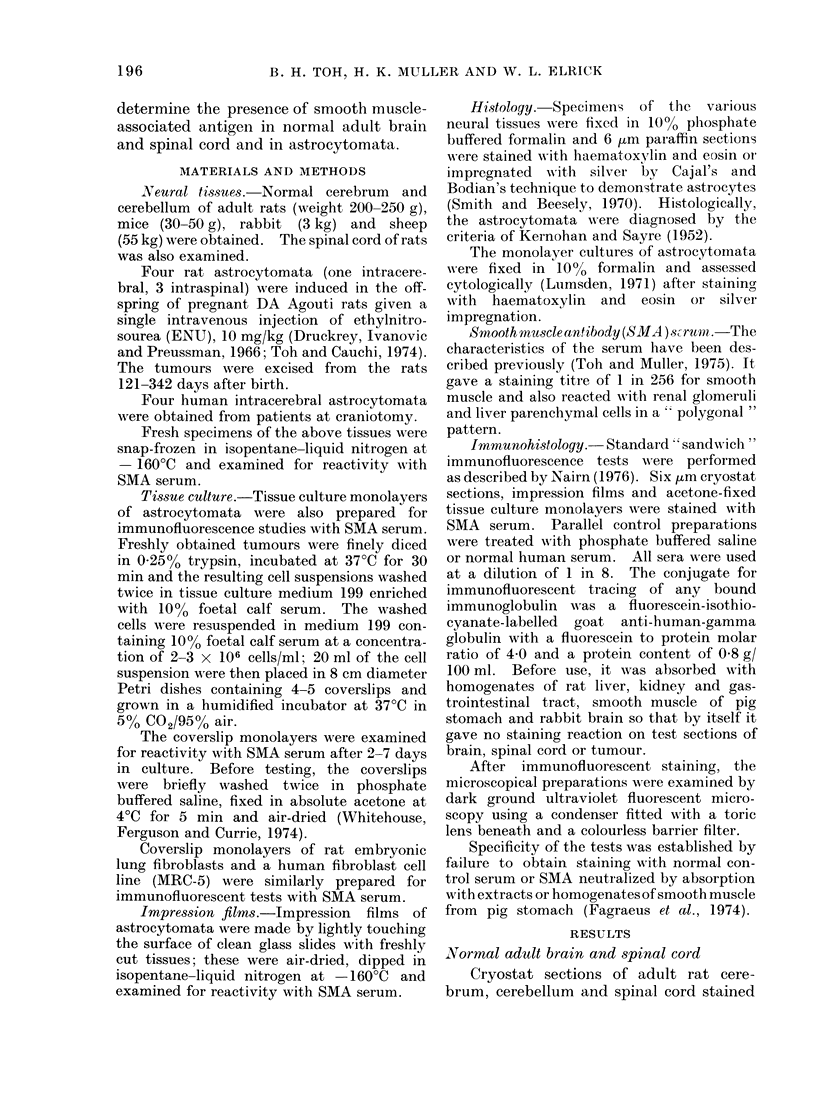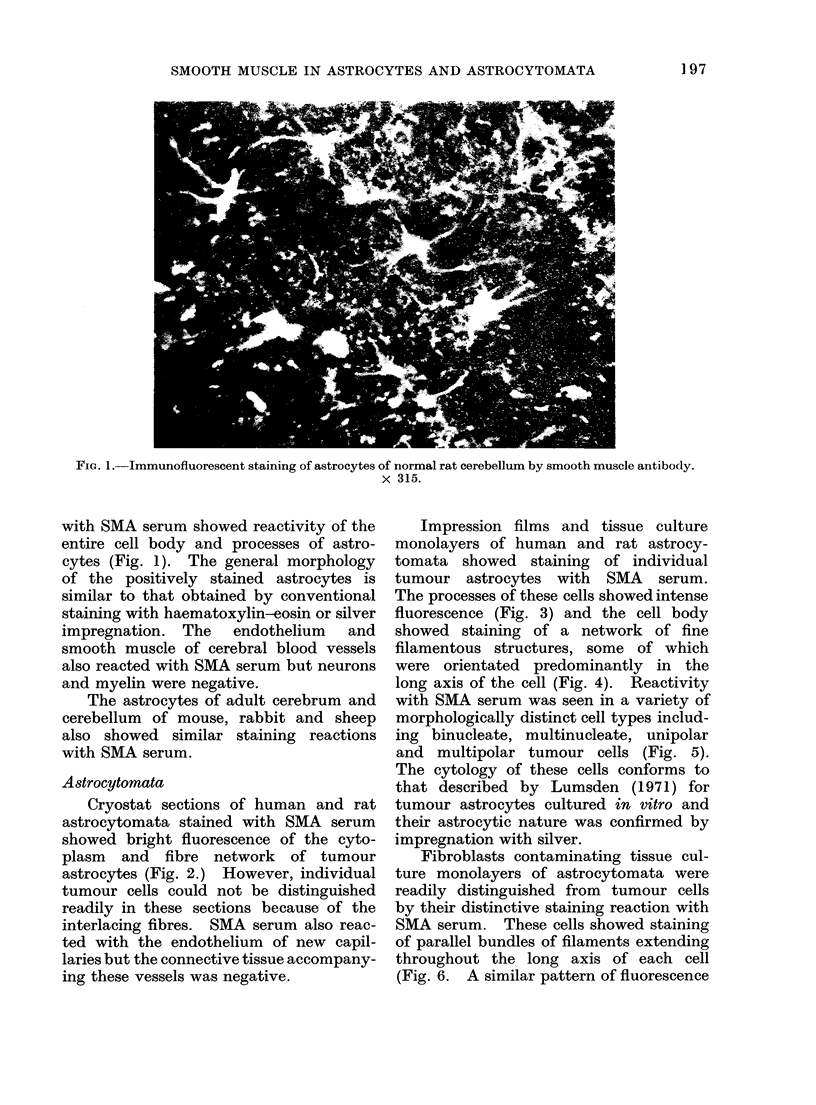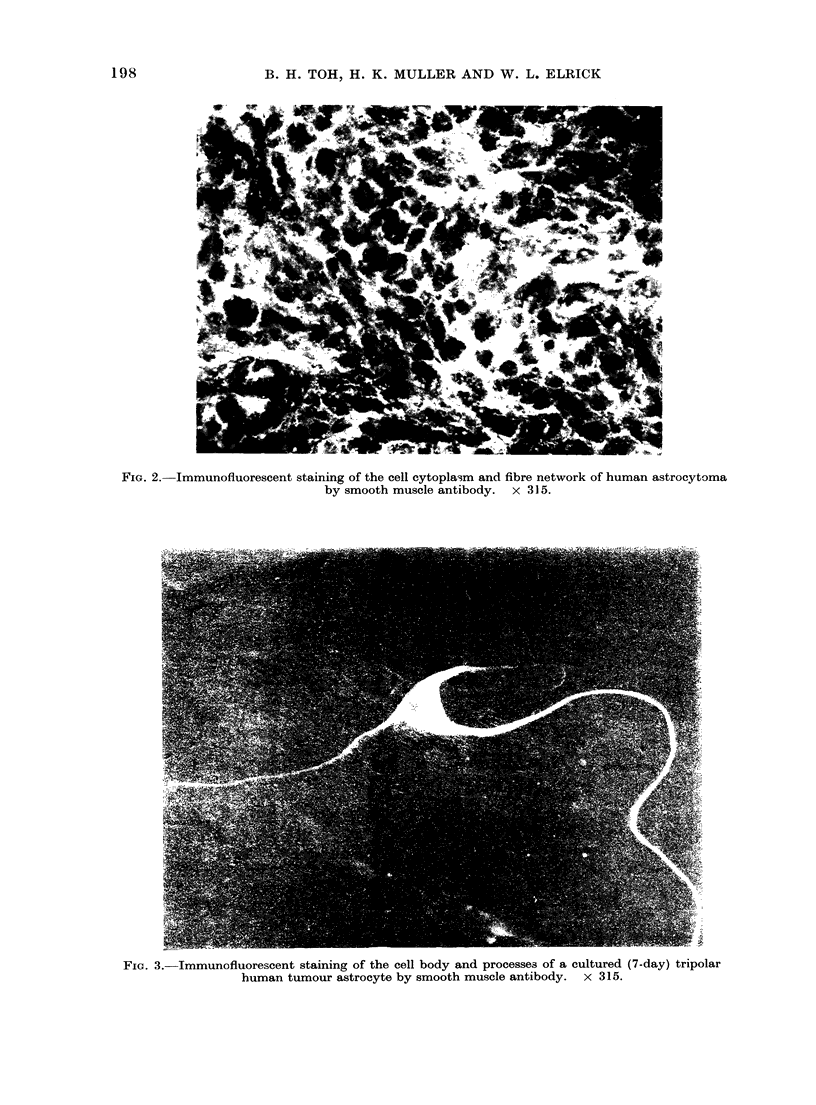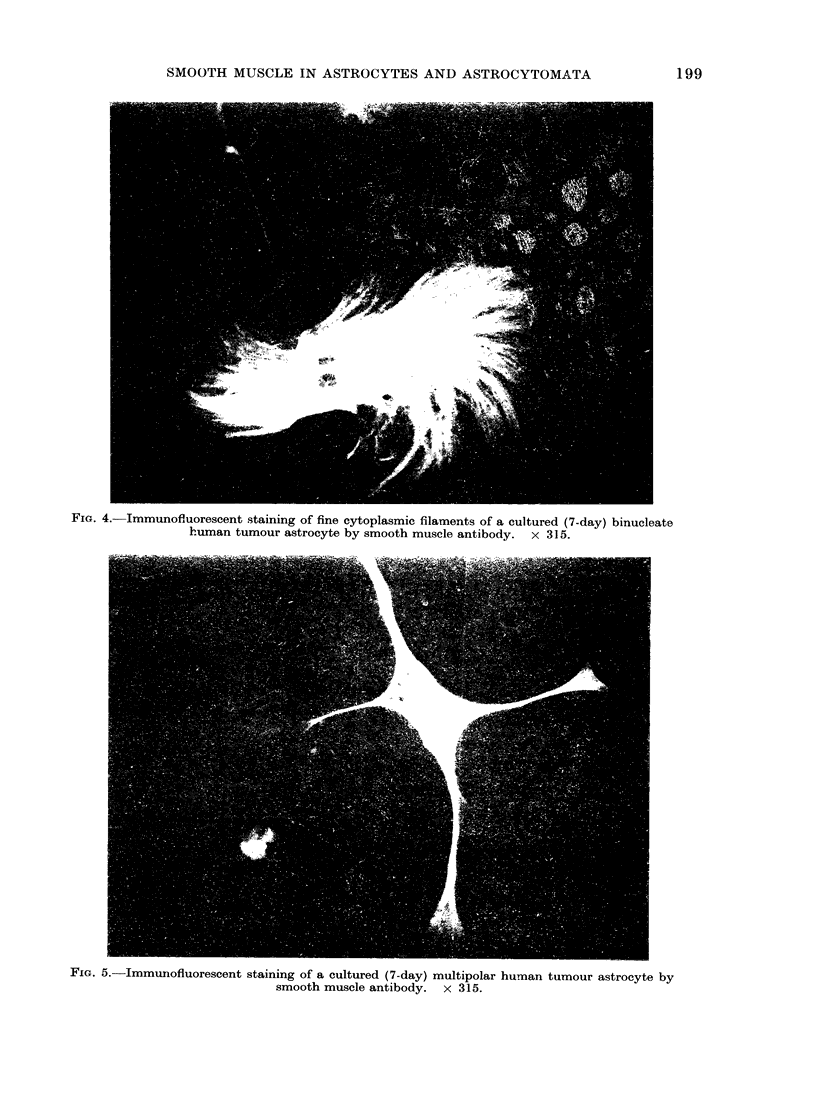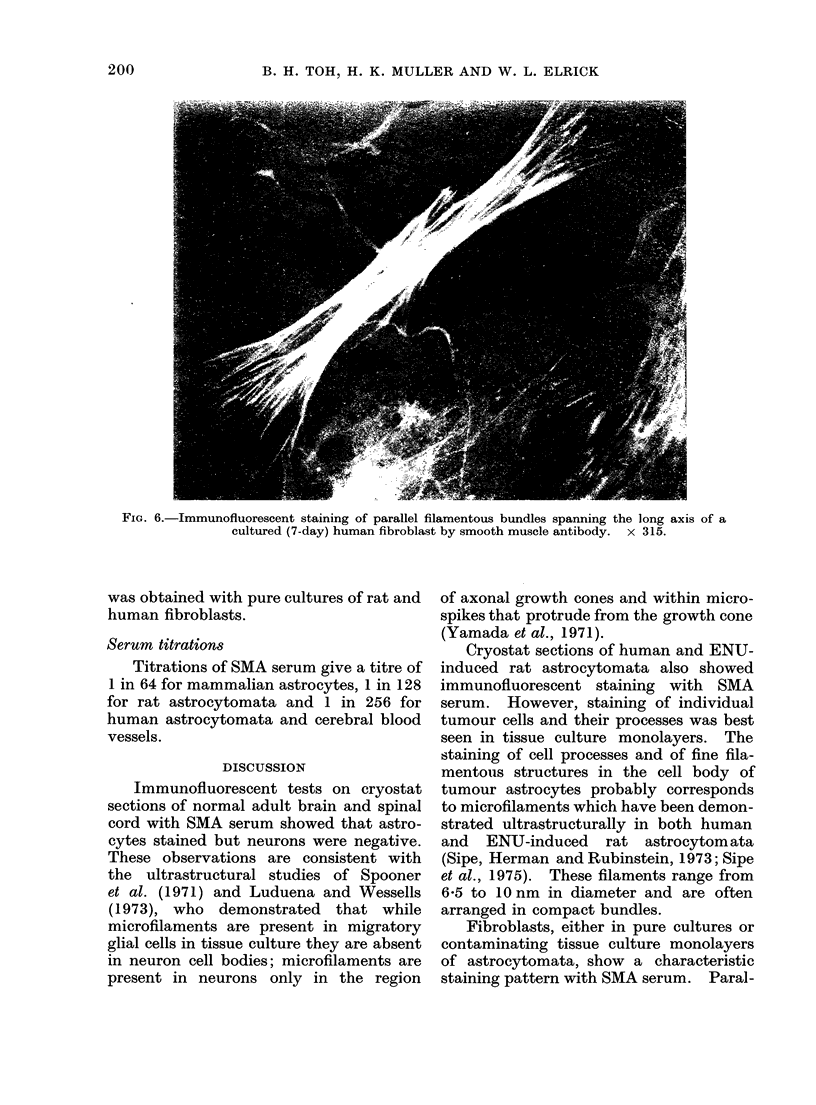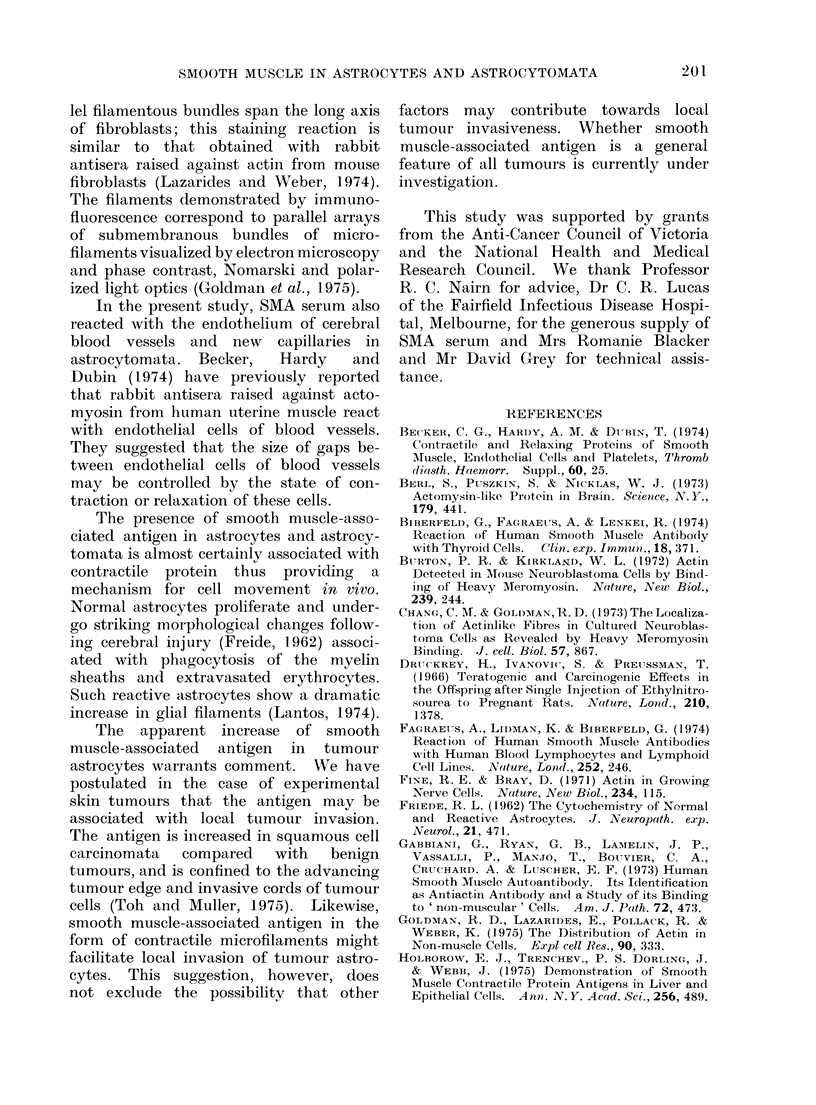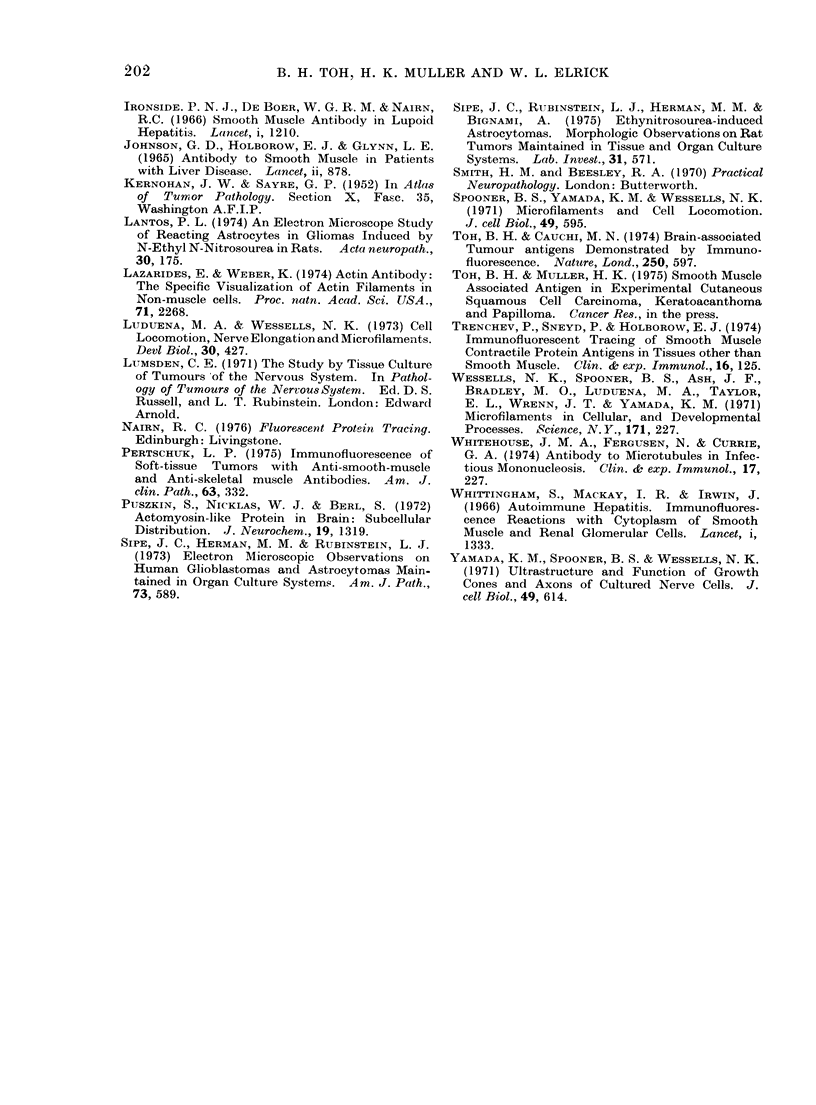# Smooth muscle associated antigen in astrocytes and astrocytomata.

**DOI:** 10.1038/bjc.1976.25

**Published:** 1976-02

**Authors:** B. H. Toh, H. K. Muller, W. L. Elrick

## Abstract

**Images:**


*Br. J. Cancer* (1976) **33**, 195

### SMOOTH MUSCLE ASSOCIATED ANTIGEN IN ASTROCYTES AND ASTROCYTOMATA

B. H. TOH*, H. K. MULLER AND W. L. ELRICK

*From the Department of Pathology and Immunology, Monash University Medical School, and Department of Neurosurgery, Alfred Hospital, Melbourne,* 3181, *Australia*

Received 9 September 1975 Accepted 20 October 1975

**Summary.—Four human and 4 rat astrocytomata and mammalian adult brain and spinal cord were examined by indirect immunofluorescence with human serum containing smooth muscle antibody. Cryostat sections of astrocytomata showed staining of the tumour cell cytoplasm and processes while in normal adult brain and spinal cord the entire astrocyte stained. Impression film and tissue culture monolayers of astrocytomata showed staining of cell processes and a fine, filamentous network in the cell body. The reaction with astrocytoma tumour cells was stronger than that with the corresponding normal astrocytes. Specificity of the staining reaction was established by its prevention on neutralization absorptions of the serum with extracts or homogeneous of smooth muscle. The presence of smooth muscle-associated antigen in astrocytes and astrocytomata is indicative of contractile protein providing a mechanism of cell movement *in vivo*.**

SERUM from patients with active chronic hepatitis reacts with smooth muscle by indirect immunofluorescence (Johnson, Holborow and Glynn, 1965; Ironside, De Boer and Nairn, 1966; Whittingham, Mackay and Irwin, 1966). Human sera containing smooth muscle antibody (SMA) also reacts with a variety of normal, " non-muscular " cells (Gabbiani *et al.*, 1973; Biberfeld, Fagraeus and Lenkei, 1974), lymphoblastoid cell lines (Fagraeus, Lidman and Biberfeld, 1974) and muscle (Pertschuk, 1975) and skin tumours (Toh and Muller, 1975).

Heterologous antisera raised against contractile proteins including actin, myosin, meromyosin and tropomyosin give patterns of staining similar to that obtained with SMA serum with the reactions abolished by absorption with the corresponding pure antigen (Trenchev, Sneyd and Holborow, 1974). Antisera to actin and meromyosin have also been demonstrated to react with microfilaments ultrastructurally (Holborow *et al.*, 1975). These observations suggest that SMA serum contains a group of antibodies to antigenically related contractile proteins associated with microfilaments and validate its use as a marker for these proteins in tissues.

Microfilaments are implicated in a number of contractile cellular phenomena (Wessells *et al.*, 1971) and have been demonstrated ultrastructurally in some neural tissues—glial cells (Spooner, Yamada and Wessells, 1971; Luduena and Wessells, 1973), the growth cone of neurite extension (Yamada, Spooner and Wessells, 1971), foetal nerve cells (Fine and Bray, 1971) and neuroblastoma (Burton and Kirkland, 1972; Chang and Goldman, 1973). An actomyosin-like protein has also been isolated from brain synaptosomal fractions (Puszkin, Nicklas and Berl, 1972) and it is suggested that if may function in the release of transmitter material at synapse endings (Berl, Puszkin and Nicklas, 1973).

The present study was undertaken to

* Dr B. H. Toh, Department of Pathology and Immunology, Monash University Medical School, Melbourne, Victoria 3181, Australia.

determine the presence of smooth muscle-associated antigen in normal adult brain and spinal cord and in astrocytomata.

### MATERIALS AND METHODS

*Neural tissues.*—Normal cerebrum and cerebellum of adult rats (weight 200–250 g), mice (30–50 g), rabbit (3 kg) and sheep (55 kg) were obtained. The spinal cord of rats was also examined.

Four rat astrocytomata (one intracerebral, 3 intraspinal) were induced in the offspring of pregnant DA Agouti rats given a single intravenous injection of ethylnitrosourea (ENU), 10 mg/kg (Druckrey, Ivanovic and Preussman, 1966; Toh and Cauchi, 1974). The tumours were excised from the rats 121–342 days after birth.

Four human intracerebral astrocytomata were obtained from patients at craniotomy.

Fresh specimens of the above tissues were snap-frozen in isopentane–liquid nitrogen at − 160°C and examined for reactivity with SMA serum.

*Tissue culture.*—Tissue culture monolayers of astrocytomata were also prepared for immunofluorescence studies with SMA serum. Freshly obtained tumours were finely diced in 0·25% trypsin, incubated at 37°C for 30 min and the resulting cell suspensions washed twice in tissue culture medium 199 enriched with 10% foetal calf serum. The washed cells were resuspended in medium 199 containing 10% foetal calf serum at a concentration of 2–3 $\times$ $10^6$ cells/ml; 20 ml of the cell suspension were then placed in 8 cm diameter Petri dishes containing 4–5 coverslips and grown in a humidified incubator at 37°C in 5% $CO_2$/95% air.

The coverslip monolayers were examined for reactivity with SMA serum after 2–7 days in culture. Before testing, the coverslips were briefly washed twice in phosphate buffered saline, fixed in absolute acetone at 4°C for 5 min and air-dried (Whitehouse, Ferguson and Currie, 1974).

Coverslip monolayers of rat embryonic lung fibroblasts and a human fibroblast cell line (MRC-5) were similarly prepared for immunofluorescent tests with SMA serum.

*Impression films.*—Impression films of astrocytomata were made by lightly touching the surface of clean glass slides with freshly cut tissues; these were air-dried, dipped in isopentane–liquid nitrogen at −160°C and examined for reactivity with SMA serum.

*Histology.*—Specimens of the various neural tissues were fixed in 10% phosphate buffered formalin and 6 μm paraffin sections were stained with haematoxylin and eosin or impregnated with silver by Cajal's and Bodian's technique to demonstrate astrocytes (Smith and Beesely, 1970). Histologically, the astrocytomata were diagnosed by the criteria of Kernohan and Sayre (1952).

The monolayer cultures of astrocytomata were fixed in 10% formalin and assessed cytologically (Lumsden, 1971) after staining with haematoxylin and eosin or silver impregnation.

*Smooth muscle antibody (SMA) serum.*—The characteristics of the serum have been described previously (Toh and Muller, 1975). It gave a staining titre of 1 in 256 for smooth muscle and also reacted with renal glomeruli and liver parenchymal cells in a " polygonal " pattern.

*Immunohistology.*— Standard "sandwich" immunofluorescence tests were performed as described by Nairn (1976). Six μm cryostat sections, impression films and acetone-fixed tissue culture monolayers were stained with SMA serum. Parallel control preparations were treated with phosphate buffered saline or normal human serum. All sera were used at a dilution of 1 in 8. The conjugate for immunofluorescent tracing of any bound immunoglobulin was a fluorescein-isothiocyanate-labelled goat anti-human-gamma globulin with a fluorescein to protein molar ratio of 4·0 and a protein content of 0·8 g/100 ml. Before use, it was absorbed with homogenates of rat liver, kidney and gastrointestinal tract, smooth muscle of pig stomach and rabbit brain so that by itself it gave no staining reaction on test sections of brain, spinal cord or tumour.

After immunofluorescent staining, the microscopical preparations were examined by dark ground ultraviolet fluorescent microscopy using a condenser fitted with a toric lens beneath and a colourless barrier filter.

Specificity of the tests was established by failure to obtain staining with normal control serum or SMA neutralized by absorption with extracts or homogenates of smooth muscle from pig stomach (Fagraeus *et al.*, 1974).

### RESULTS

### *Normal adult brain and spinal cord*

Cryostat sections of adult rat cerebrum, cerebellum and spinal cord stained

FIG. 1.—Immunofluorescent staining of astrocytes of normal rat cerebellum by smooth muscle antibody. × 315.

with SMA serum showed reactivity of the entire cell body and processes of astrocytes (Fig. 1). The general morphology of the positively stained astrocytes is similar to that obtained by conventional staining with haematoxylin–eosin or silver impregnation. The endothelium and smooth muscle of cerebral blood vessels also reacted with SMA serum but neurons and myelin were negative.

The astrocytes of adult cerebrum and cerebellum of mouse, rabbit and sheep also showed similar staining reactions with SMA serum.

*Astrocytomata*

Cryostat sections of human and rat astrocytomata stained with SMA serum showed bright fluorescence of the cytoplasm and fibre network of tumour astrocytes (Fig. 2.) However, individual tumour cells could not be distinguished readily in these sections because of the interlacing fibres. SMA serum also reacted with the endothelium of new capillaries but the connective tissue accompanying these vessels was negative.

Impression films and tissue culture monolayers of human and rat astrocytomata showed staining of individual tumour astrocytes with SMA serum. The processes of these cells showed intense fluorescence (Fig. 3) and the cell body showed staining of a network of fine filamentous structures, some of which were orientated predominantly in the long axis of the cell (Fig. 4). Reactivity with SMA serum was seen in a variety of morphologically distinct cell types including binucleate, multinucleate, unipolar and multipolar tumour cells (Fig. 5). The cytology of these cells conforms to that described by Lumsden (1971) for tumour astrocytes cultured *in vitro* and their astrocytic nature was confirmed by impregnation with silver.

Fibroblasts contaminating tissue culture monolayers of astrocytomata were readily distinguished from tumour cells by their distinctive staining reaction with SMA serum. These cells showed staining of parallel bundles of filaments extending throughout the long axis of each cell (Fig. 6. A similar pattern of fluorescence

FIG. 2.—Immunofluorescent staining of the cell cytoplasm and fibre network of human astrocytoma by smooth muscle antibody. × 315.

FIG. 3.—Immunofluorescent staining of the cell body and processes of a cultured (7-day) tripolar human tumour astrocyte by smooth muscle antibody. × 315.

FIG. 4.—Immunofluorescent staining of fine cytoplasmic filaments of a cultured (7-day) binucleate human tumour astrocyte by smooth muscle antibody. × 315.

FIG. 5.—Immunofluorescent staining of a cultured (7-day) multipolar human tumour astrocyte by smooth muscle antibody. × 315.

FIG. 6.—Immunofluorescent staining of parallel filamentous bundles spanning the long axis of a cultured (7-day) human fibroblast by smooth muscle antibody. × 315.

was obtained with pure cultures of rat and human fibroblasts.

*Serum titrations*

Titrations of SMA serum give a titre of 1 in 64 for mammalian astrocytes, 1 in 128 for rat astrocytomata and 1 in 256 for human astrocytomata and cerebral blood vessels.

DISCUSSION

Immunofluorescent tests on cryostat sections of normal adult brain and spinal cord with SMA serum showed that astrocytes stained but neurons were negative. These observations are consistent with the ultrastructural studies of Spooner *et al.* (1971) and Luduena and Wessells (1973), who demonstrated that while microfilaments are present in migratory glial cells in tissue culture they are absent in neuron cell bodies; microfilaments are present in neurons only in the region of axonal growth cones and within microspikes that protrude from the growth cone (Yamada *et al.*, 1971).

Cryostat sections of human and ENU-induced rat astrocytomata also showed immunofluorescent staining with SMA serum. However, staining of individual tumour cells and their processes was best seen in tissue culture monolayers. The staining of cell processes and of fine filamentous structures in the cell body of tumour astrocytes probably corresponds to microfilaments which have been demonstrated ultrastructurally in both human and ENU-induced rat astrocytomata (Sipe, Herman and Rubinstein, 1973; Sipe *et al.*, 1975). These filaments range from 6·5 to 10 nm in diameter and are often arranged in compact bundles.

Fibroblasts, either in pure cultures or contaminating tissue culture monolayers of astrocytomata, show a characteristic staining pattern with SMA serum. Parallel filamentous bundles span the long axis of fibroblasts; this staining reaction is similar to that obtained with rabbit antisera raised against actin from mouse fibroblasts (Lazarides and Weber, 1974). The filaments demonstrated by immunofluorescence correspond to parallel arrays of submembranous bundles of microfilaments visualized by electron microscopy and phase contrast, Nomarski and polarized light optics (Goldman *et al.*, 1975).

In the present study, SMA serum also reacted with the endothelium of cerebral blood vessels and new capillaries in astrocytomata. Becker, Hardy and Dubin (1974) have previously reported that rabbit antisera raised against actomyosin from human uterine muscle react with endothelial cells of blood vessels. They suggested that the size of gaps between endothelial cells of blood vessels may be controlled by the state of contraction or relaxation of these cells.

The presence of smooth muscle-associated antigen in astrocytes and astrocytomata is almost certainly associated with contractile protein thus providing a mechanism for cell movement *in vivo*. Normal astrocytes proliferate and undergo striking morphological changes following cerebral injury (Freide, 1962) associated with phagocytosis of the myelin sheaths and extravasated erythrocytes. Such reactive astrocytes show a dramatic increase in glial filaments (Lantos, 1974).

The apparent increase of smooth muscle-associated antigen in tumour astrocytes warrants comment. We have postulated in the case of experimental skin tumours that the antigen may be associated with local tumour invasion. The antigen is increased in squamous cell carcinomata compared with benign tumours, and is confined to the advancing tumour edge and invasive cords of tumour cells (Toh and Muller, 1975). Likewise, smooth muscle-associated antigen in the form of contractile microfilaments might facilitate local invasion of tumour astrocytes. This suggestion, however, does not exclude the possibility that other factors may contribute towards local tumour invasiveness. Whether smooth muscle-associated antigen is a general feature of all tumours is currently under investigation.

This study was supported by grants from the Anti-Cancer Council of Victoria and the National Health and Medical Research Council. We thank Professor R. C. Nairn for advice, Dr C. R. Lucas of the Fairfield Infectious Disease Hospital, Melbourne, for the generous supply of SMA serum and Mrs Romanie Blacker and Mr David Grey for technical assistance.

REFERENCES

Becker, C. G., Hardy, A. M. & Dubin, T. (1974) Contractile and Relaxing Proteins of Smooth Muscle, Endothelial Cells and Platelets, *Thromb diasth. Haemorr.* Suppl., **60**, 25.

Berl, S., Puszkin, S. & Nicklas, W. J. (1973) Actomysin-like Protein in Brain. *Science, N.Y.*, **179**, 441.

Biberfeld, G., Fagraeus, A. & Lenkei, R. (1974) Reaction of Human Smooth Muscle Antibody with Thyroid Cells. *Clin. exp. Immun.*, **18**, 371.

Burton, P. R. & Kirkland, W. L. (1972) Actin Detected in Mouse Neuroblastoma Cells by Binding of Heavy Meromyosin. *Nature, New Biol.*, **239**, 244.

Chang, C. M. & Goldman, R. D. (1973) The Localization of Actinlike Fibres in Cultured Neuroblastoma Cells as Revealed by Heavy Meromyosin Binding. *J. cell. Biol.* **57**, 867.

Druckrey, H., Ivanovic, S. & Preussman, T. (1966) Teratogenic and Carcinogenic Effects in the Offspring after Single Injection of Ethylnitrosourea to Pregnant Rats. *Nature, Lond.*, **210**, 1378.

Fagraeus, A., Lidman, K. & Biberfeld, G. (1974) Reaction of Human Smooth Muscle Antibodies with Human Blood Lymphocytes and Lymphoid Cell Lines. *Nature, Lond.*, **252**, 246.

Fine, R. E. & Bray, D. (1971) Actin in Growing Nerve Cells. *Nature, New Biol.*, **234**, 115.

Friede, R. L. (1962) The Cytochemistry of Normal and Reactive Astrocytes. *J. Neuropath. exp. Neurol.*, **21**, 471.

Gabbiani, G., Ryan, G. B., Lamelin, J. P., Vassalli, P., Manjo, T., Bouvier, C. A., Cruchard. A. & Luscher, E. F. (1973) Human Smooth Muscle Autoantibody. Its Identification as Antiactin Antibody and a Study of its Binding to ' non-muscular ' Cells. *Am. J. Path.* **72**, 473.

Goldman, R. D., Lazarides, E., Pollack, R. & Weber, K. (1975) The Distribution of Actin in Non-muscle Cells. *Expl cell Res.*, **90**, 333.

Holborow, E. J., Trenchev., P. S. Dorling, J. & Webb, J. (1975) Demonstration of Smooth Muscle Contractile Protein Antigens in Liver and Epithelial Cells. *Ann. N.Y. Acad. Sci.*, **256**, 489.

IRONSIDE. P. N. J., DE BOER, W. G. R. M. & NAIRN, R.C. (1966) Smooth Muscle Antibody in Lupoid Hepatitis. *Lancet*, i, 1210.

JOHNSON, G. D., HOLBOROW, E. J. & GLYNN, L. E. (1965) Antibody to Smooth Muscle in Patients with Liver Disease. *Lancet*, ii, 878.

KERNOHAN, J. W. & SAYRE, G. P. (1952) In *Atlas of Tumor Pathology*. Section X, Fasc. 35, Washington A.F.I.P.

LANTOS, P. L. (1974) An Electron Microscope Study of Reacting Astrocytes in Gliomas Induced by N-Ethyl N-Nitrosourea in Rats. *Acta neuropath.*, **30**, 175.

LAZARIDES, E. & WEBER, K. (1974) Actin Antibody: The Specific Visualization of Actin Filaments in Non-muscle cells. *Proc. natn. Acad. Sci. USA.*, **71**, 2268.

LUDUENA, M. A. & WESSELLS, N. K. (1973) Cell Locomotion, Nerve Elongation and Microfilaments. *Devl Biol.*, **30**, 427.

LUMSDEN, C. E. (1971) The Study by Tissue Culture of Tumours of the Nervous System. In *Pathology of Tumours of the Nervous System*. Ed. D. S. Russell, and L. T. Rubinstein. London: Edward Arnold.

NAIRN, R. C. (1976) *Fluorescent Protein Tracing*. Edinburgh: Livingstone.

PERTSCHUK, L. P. (1975) Immunofluorescence of Soft-tissue Tumors with Anti-smooth-muscle and Anti-skeletal muscle Antibodies. *Am. J. clin. Path.*, **63**, 332.

PUSZKIN, S., NICKLAS, W. J. & BERL, S. (1972) Actomyosin-like Protein in Brain: Subcellular Distribution. *J. Neurochem.*, **19**, 1319.

SIPE, J. C., HERMAN, M. M. & RUBINSTEIN, L. J. (1973) Electron Microscopic Observations on Human Glioblastomas and Astrocytomas Maintained in Organ Culture Systems. *Am. J. Path.*, **73**, 589.

SIPE, J. C., RUBINSTEIN, L. J., HERMAN, M. M. & BIGNAMI, A. (1975) Ethynitrosourea-induced Astrocytomas. Morphologic Observations on Rat Tumors Maintained in Tissue and Organ Culture Systems. *Lab. Invest.*, **31**, 571.

SMITH, H. M. and BEESLEY, R. A. (1970) *Practical Neuropathology*. London: Butterworth.

SPOONER, B. S., YAMADA, K. M. & WESSELLS, N. K. (1971) Microfilaments and Cell Locomotion. *J. cell Biol.*, **49**, 595.

TOH, B. H. & CAUCHI, M. N. (1974) Brain-associated Tumour antigens Demonstrated by Immunofluorescence. *Nature, Lond.*, **250**, 597.

TOH, B. H. & MULLER, H. K. (1975) Smooth Muscle Associated Antigen in Experimental Cutaneous Squamous Cell Carcinoma, Keratoacanthoma and Papilloma. *Cancer Res.*, in the press.

TRENCHEV, P., SNEYD, P. & HOLBOROW, E. J. (1974) Immunofluorescent Tracing of Smooth Muscle Contractile Protein Antigens in Tissues other than Smooth Muscle. *Clin. & exp. Immunol.*, **16**, 125.

WESSELLS, N. K., SPOONER, B. S., ASH, J. F., BRADLEY, M. O., LUDUENA, M. A., TAYLOR, E. L., WRENN, J. T. & YAMADA, K. M. (1971) Microfilaments in Cellular, and Developmental Processes. *Science, N.Y.*, **171**, 227.

WHITEHOUSE, J. M. A., FERGUSEN, N. & CURRIE, G. A. (1974) Antibody to Microtubules in Infectious Mononucleosis. *Clin. & exp. Immunol.*, **17**, 227.

WHITTINGHAM, S., MACKAY, I. R. & IRWIN, J. (1966) Autoimmune Hepatitis. Immunofluorescence Reactions with Cytoplasm of Smooth Muscle and Renal Glomerular Cells. *Lancet*, i, 1333.

YAMADA, K. M., SPOONER, B. S. & WESSELLS, N. K. (1971) Ultrastructure and Function of Growth Cones and Axons of Cultured Nerve Cells. *J. cell Biol.*, **49**, 614.